# Version VI of the ESTree db: an improved tool for peach transcriptome analysis

**DOI:** 10.1186/1471-2105-9-S2-S9

**Published:** 2008-03-26

**Authors:** Barbara Lazzari, Andrea Caprera, Alberto Vecchietti, Ivan Merelli, Francesca Barale, Luciano Milanesi, Alessandra Stella, Carlo Pozzi

**Affiliations:** 1 Parco Tecnologico Padano, Via Einstein - Località Cascina Codazza, Lodi, 26900, Italy; 2 Istituto Tecnologie Biomediche, CNR, Via Fratelli Cervi 93, Segrate (MI), 20090, Italy; 3 Università degli Studi di Milano, Facoltà di Scienze Agrarie, via Celoria 2, Milan, 20133, Italy

## Abstract

**Background:**

The ESTree database (db) is a collection of *Prunus persica* and *Prunus dulcis* EST sequences that in its current version encompasses 75,404 sequences from 3 almond and 19 peach libraries. Nine peach genotypes and four peach tissues are represented, from four fruit developmental stages. The aim of this work was to implement the already existing ESTree db by adding new sequences and analysis programs. Particular care was given to the implementation of the web interface, that allows querying each of the database features.

**Results:**

A Perl modular pipeline is the backbone of sequence analysis in the ESTree db project. Outputs obtained during the pipeline steps are automatically arrayed into the fields of a MySQL database. Apart from standard clustering and annotation analyses, version VI of the ESTree db encompasses new tools for tandem repeat identification, annotation against genomic Rosaceae sequences, and positioning on the database of oligomer sequences that were used in a peach microarray study. Furthermore, known protein patterns and motifs were identified by comparison to PROSITE. Based on data retrieved from sequence annotation against the UniProtKB database, a script was prepared to track positions of homologous hits on the GO tree and build statistics on the ontologies distribution in GO functional categories. EST mapping data were also integrated in the database. The PHP-based web interface was upgraded and extended. The aim of the authors was to enable querying the database according to all the biological aspects that can be investigated from the analysis of data available in the ESTree db. This is achieved by allowing multiple searches on logical subsets of sequences that represent different biological situations or features.

**Conclusions:**

The version VI of ESTree db offers a broad overview on peach gene expression. Sequence analyses results contained in the database, extensively linked to external related resources, represent a large amount of information that can be queried via the tools offered in the web interface. Flexibility and modularity of the ESTree analysis pipeline and of the web interface allowed the authors to set up similar structures for different datasets, with limited manual intervention.

## Background

The ESTree db [1] is an Expressed Sequence Tags (ESTs) database that was developed by the Italian ESTree Interuniversitary Centre as a platform for easy genomics and functional genomics data integration and retrieval. Together with the GDR database [2], it represents the most complete online resource for peach EST analysis. The ESTree db sequence analysis is based on a semi-automated Perl pipeline that during its steps feeds the tables of a MySQL database. Queries to the database can be performed via a PHP-based web interface.

The first version of the ESTree db released in 2004 encompassed a restricted number of peach sequences, derived from four peach mesocarp in-house prepared libraries. In the following versions, public peach sequences were added to the collection, and in version III (released on April 2005) [3] the number of represented libraries was grown to eight. Further versions of the database were released in the past three years, each of which encompasses more sequences and more features. In the currently released version VI (as of March 2007), the collection has grown to 75,404 sequences: 10,847 derived from five in-house prepared libraries from peach fruits and the others downloaded from GenBank or kindly provided by other members of the ESTree Interuniversitary Centre. The database is mostly devoted to *Prunus persica*, but 3,864 *Prunus dulcis* sequences (i.e. the almond ESTs currently publicly available) were also added. The resulting dataset was composed of sequences obtained from twenty-two libraries (three from almond and nineteen from peach), seven of which were added to Version VI for the first time. The peach dataset, in particular, represented nine genotypes and four tissues, and mesocarp sequences were obtained from the four developmental stages describing the peach ripening process. The availability of a more extended sequence dataset allowed the authors to explore in more detail the differences in gene expression in the various tissues and developmental stages, making use of an extended version of the ESTree db pipeline that integrates a more comprehensive collection of sequence analysis programs.

The aim of this work is to describe the major changes and improvements brought to the ESTree db, and to inform users of the availability of this extended tool, for an easy exploration of peach and related species functional genomics.

## Construction and content

### The ESTree db sequence collection

The ESTree db encompasses peach and almond sequences. The few available almond sequences were introduced in the database to support mapping data produced on the TxE Prunus reference map based on an almond x peach F2 progeny. Almond sequences downloaded from GenBank were from pistils and embryo, plus a third library of unknown origin. The collection of peach sequences represents nine genotypes (Bolero, Loring, OroA, Red Haven, Suncrest, Yumeyong, O'Henry, Baby gold 5 and Fantasia), four tissues (mesocarp, skin, shoot, and leaf) and four mesocarp developmental stages (post-allegation, pit hardening, pre-climacteric, and post-climacteric). Distribution of sequences among libraries or developmental stages was unequal, the mesocarp post-climateric stage being the most represented. In program outputs displaying sequences from different libraries (i.e. contigs graphical displays or outputs from the putative SNP identification procedure), a colour code was assigned to relate sequences to the tissue and developmental stage of origin. Sequence names themselves were library-declarative, as library identifiers were used as sequence name prefixes.

### Sequence analysis pipeline and database structures

**Figure 1:**
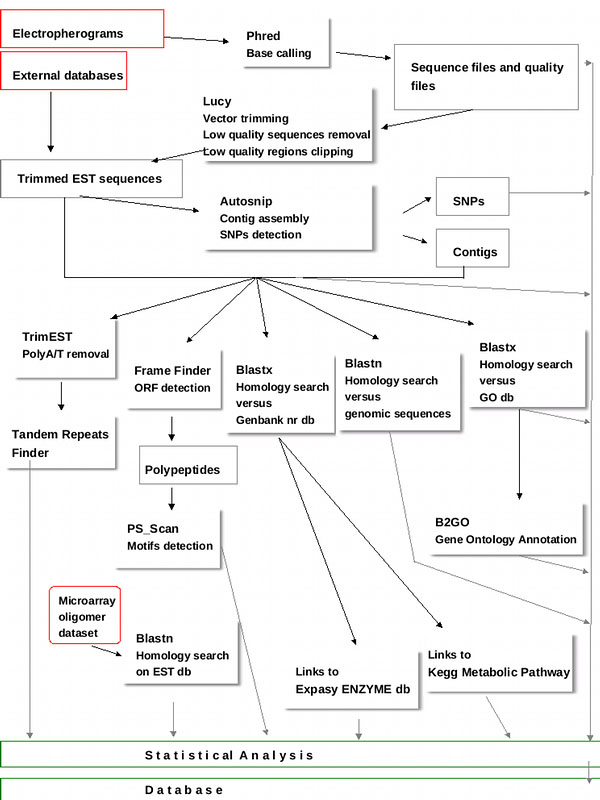
**The ESTree db pipeline.** The main programs constituting the pipeline scheme. Accessory scripts are not presented in the diagram.

The ESTree sequence analysis pipeline (Figure 1) has a modular structure suitable for adaptation to different datasets. Similar pipelines, in fact, were used by the authors in other projects, with different datasets [4,5]. The backbone of the pipeline remained as described in Lazzari *et al*., 2005 [3], but new modules were added to accomplish further analyses and existing modules were partially changed to meet new requirements. A full description of programs that were integrated in the pipeline and parameters settings is given at the “Processing, Assembly and Annotation” page of the web site.

The MySQL database where data are stored has expanded to contain all the new programs' outputs and all the tables that are queried to produce dynamic outputs upon users' requests.

### Sequence clustering and putative SNP detection

The ESTree db pipeline input file was the multifasta file containing the complete dataset of 19 libraries. Electropherogram reading, vector clipping and poor region removal were performed with Phred [6] and Lucy [7] (parameters: max_avg_error 0.025 max_error_at_ends 0.02) on in-house produced sequences. These last programs were not included in the pipeline because chromatograms of sequences downloaded from GenBank were not available. The dishomogeneity in terms of the quality information forced us to adopt putative SNP detection programs where quality data were not mandatory. The AutoSNP algorithm [8], version 7, was chosen for this purpose. In the former ESTree db versions, the assembly and the putative SNP detection procedures were carried on independently, with CAP3 [9] and AutoSNP, respectively. As a result, a non-matching nomenclature between the contig sets generated by the two procedures was observed. In version VI, both the procedures were carried out using the AutoSNP program. Perl scripts were prepared to extract assembly data from the intermediate steps of the AutoSNP procedure. The contrasting nomenclature problems were overcome by exploiting AutoSNP capability of integrating TGICL [10] and CAP3 to prepare sequence assemblies before the actual putative SNP detection algorithm is run. With TGICL parameters set to -p 95, -l 60 and -v 20 and CAP 3 parameters set to -p 98, -o 100 and -f 30, the 75,404 sequences of the ESTree db were assembled in 7,709 contigs and 20,682 singletons, representing a unigene dataset composed of 28,391 sequences (37.6% of the total sequences) (Table 1).

**Table 1:** The ESTree db statistics on sequences. Comparison of the main statistics on the ESTree db version VI, compared with the previously described version III.

	**ESTree III**	**ESTree VI**
Total nr of seq	18,630	75,404
Total nr of contigs	2,328	7,709
Average alignment depth	5	7.1
Total nr of singletons	6,891	20,682
Total nr of unigenes	9,219	28,391
In-house produced seq	6,157	10,847
Total almond seq	-	3,864
Total peach seq	18,630	71,540
*Pp* mesocarp seq	18,630	55,996
*Pp* skin seq	-	4,690
*Pp* shoot seq	-	7,085
*Pp* leaf seq	-	3,769
NCBI annotated seq (%)	70.4	78.6
GO annotated seq (%)	49.9	69.7
Genomic annotated seq (%)	-	26.8
Repeats-containing seq (%)	-	16
Putative SNP-containing seq (%)	12.7	22.9
PROSITE patterns-containing seq (%)	-	18
Mapped sequences	-	0.75
Seq associated to microarray data (%)	-	6.2
Metabolic pathways-associated seq (%)	1,8	0.62

### Changes and improvements of the ESTree db annotation procedures

In the ESTree db, version VI, ESTs and contig consensus sequences were annotated against three different databases, with an E-value cutoff of ≤ ^e−10^. The master annotation was performed by BLASTx against the GenBank nr db, in order to retrieve the highest number of annotations; a second annotation was performed by BLASTn against a custom-prepared database composed of 7,829 Rosaceae genomic DNA/RNA sequences downloaded from the GenBank CoreNucleotide db in October 2006. The already existing so-called “GO annotation” procedure - aimed to the identification of proteins homologs annotated according to the Gene Ontology (GO) project [11,12] - was adapted to specific needs. In the preceding ESTree db versions, in fact, GO annotation was performed online, and not integrated into the ESTree pipeline, using the GOblet [13] program against the Viridiplantae subset of the GO database (a subset of sp-trembl). The number of sequences that had to be annotated, the need for frequent annotation updates, and the requirement to perform a more extensive annotation against the whole UniProtKB database, called for the improvement produced in version VI. A number of Perl scripts were prepared to create a table with all the protein-GO associations including a no-direct link due to “is_a” relations among different GO elements, starting from information contained in the GoA association file as well as in the GO DAG (Directed Acyclic Graph) file. Information contained in this table was used to produce statistics on the ontologies distribution that can be browsed on all the GO tree levels.

According to the adopted E-value cutoff, 78.6% of the sequences contained in the ESTree db were annotated against the GenBank nr db, 69.7% against the UniProtKB db, and 26.8% against the genomic Rosaceae db (Table 1).

Considering the high number of sequences that had to be annotated and the need to perform annotation against there different databases, a parallel approach was adopted to perform BLAST searches on a computer cluster [14], giving the possibility to retrieve results in reasonable times. When blast outputs are recovered, they are parsed, split in single-query files and stored in MySQL tables as such and in feature-dedicated fields.

### GO statistics

**Figure 2:**
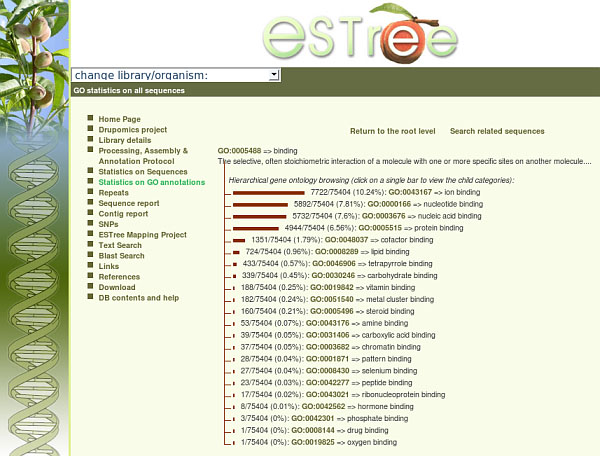
**GO Statistics in the ESTree db**. An example of a GO Statistics page in the ESTree db. Clicking on bars allows hierarchical ontologies browsing. Sequences matching each GO identifier are retrievable via the “Search related sequences” link.

The GO DAG was the source of information from which paths of GO identifiers were retrieved for each significantly annotated sequence: the global statistics were created using each found identifier to increase GO category-specific counters. In the “Statistics on GO annotations” page of the web interface (Figure 2), GO statistics on all sequences, as well as the percentage of un-annotated sequences, are given. Sequences matching the different categories are presented as proportional bars, which are dynamically created by the PHP-based web interface. Hierarchical browsing of ontologies is allowed. Via the “Change library/organism” box of the “Statistics on GO annotations” web page, statistics on ontologies distributions related to specific sequence subsets can be accessed. In total, 21 sequence sets are available, allowing ontologies browsing on sequences from single libraries, as well as on sequences from biologically relevant datasets, made homogeneous with respect to the tissue of origin and/or the developmental stage. Peach and almond sequences can be considered as a whole set or independently. Retrieval of ESTree sequences matching each GO class or sub-class is also possible by clicking on “Search related sequences” at the top of each statistics page. Percentages of sequences matching each GO category are given near each bar.

### Tandem repeats search

A repeat analysis feature was introduced in the ESTree db and the Tandem Repeats Finder program [15] was used for repeat identification. Prior to repeat identification, PolyA and PolyT tails were trimmed from sequences with TrimEST [16]. The Tandem Repeats Finder algorithm locates and displays two or more adjacent, approximate copies of a pattern of nucleotides in DNA sequences. With the selected settings (2 7 7 80 10 50 1000), the program was able to identify repeats of variable period size (from single base stretches to very long base sequences) that were encountered in more than one copy within 1,000 base pairs. This allowed the identification of simple sequence repeats (SSR, with period size from 2 to 6 base pairs) as well as of longer and more dispersed tandem repeats that are present in the submitted sequences. The MISA software [17] was applied to the same dataset, and analogous results were retrieved (data not shown). In total, 12,068 repeat-containing sequences were identified in the ESTree collection, and among these 3,946 (32.7%) were SSRs.

In order to allow users to choose sets of repeats of given characteristics (i.e. intervals of period size and copy number), a search engine was prepared.

### The ESTree mapping project

The aim of the ESTree Mapping Project is to map approximately 200 ESTs on a genetic map [18], as a prerequisite for further positioning on a physical framework map based on BAC clones fingerprinted at Clemson University [2]. The preliminary mapping results were presented in the ESTree db, together with the peach ESTs map positions that were publicly available at the GDR web site. Currently, mapping information is available for 568 sequences. In the Sequence Report page, map information was linked to the CMap pages of the GDR.

A program that allows the automatic BIN assignment based on a TxE subset of plants [19] was prepared, and can either be used online or downloaded via the links that are given in the “ESTree Mapping Project” page.

### Analyses on the inferred protein dataset

The ESTate package programs [20] were used to deduce from each EST and contig consensus a putative protein sequence. FrameFinder uses log odds hexamer frequency statistics and multiple dynamic programming frame-shifts models to predict the reading frame of CDSs, and, being error-tolerant, is particularly suitable for the analysis of EST sequences. To create log odds probabilities for FrameFinder, a training set, composed of Rosaceae mRNA sequences downloaded from the NCBI CoreNucleotide database was prepared and processed with fasta2usage (parameters: -w 6 -j 3) and wordprob. The FrameFinder output, encompassing the most probable putative protein sequence that was obtained from each input sequence, was compared to the PROSITE database [21] using the software scanPROSITE [22], and matching patterns were stored in the database. Frequently matching patterns and profiles were excluded from the analysis. In total, PROSITE patterns and domains were identified in 13,580 EST sequences and in 1,846 contig consensus sequences.

### Upgrading the web interface

The PHP-based web interface was upgraded and modified in Version VI. The Sequence Report and Contig Report tables represent the standard format for data presentation in the ESTree db, and query outputs or selectable sequence subsets are presented with the same table structure. To integrate new data, more columns were added to the tables, so that each sequence feature is presented in table fields and linked to dedicated pages, internal to the ESTree db or belonging to related web resources. A column was added to associate the 4,699 oligomer sequences used in a peach microarray study [23] to the correspondent sequences contained in the database. This tabular structure allows complete overview of sequence-associated features simply by scrolling the tables. Query outputs can also be downloaded, both in tabular format or as multifasta file, encompassing sequences matching the query terms. Independent browsing of subsets of sequences was allowed. This is crucial for a deep insight in the different biological situations existing in the tissues and developmental stages that are represented in the database. Sequences can be partitioned according to the organism (peach or almond), the library, the tissue or developmental stage, whether they belong to a unigene, singlet or contig, the presence of repeats or putative SNPs and of map positions. In the “Download” page, download was allowed in multiple formats for EST sequences and contig consensus sequences, as well as for the CAP3 alignment and .ace files and for the AutoSNP SNP Reports.

## Utility and discussion

The ESTree and the GDR databases represent the only existing online resources dedicated to peach EST analysis. The two databases are very similar in terms of entry number (71,540 peach sequences in the ESTree db, 70,939 in the GDR db), but quite different in terms of information and its retrieval. The ESTree db clustering procedure produced a dataset of 27,097 unigenes, 4,303 of which were derived from our in-house prepared libraries.

From the bioinformatic point of view, flexibility and modularity were the keywords in structuring the database. The authors prepared an EST analysis pipeline that could be conveniently applied to the analysis of data derived from other projects. The ESTree pipeline invokes standard public programs as well as custom prepared Perl modules. Adding or removing modules requires little manual intervention, making the pipeline easily adaptable to the specific requirements of different datasets. Nonetheless, because of the progressive introduction of new data and of the significant amount of data updating needed to compile the database tables, an effort was made to reduce the time required to complete the whole procedure. BLAST analyses were not included into the pipeline to allow a parallel approach on a computer cluster. Updates in the GO blast procedure call for the updating of the corresponding in-house prepared table upon which GO statistics are created, and this is another example of time-consuming task that is carried out independently from the main pipeline. In the whole ESTree db structure, the PHP-based web interface plays a fundamental role. All the graphical outputs that are presented in the web site are prepared on-the-fly by this interface, and are not stored in the database.

The ESTree project is still underway, and additional data are being produced. The integration of microarray-derived data and of EST frequency analyses in different tissues and developmental stages into the ESTree db will make it an exhaustive tool for interpretation of expression differences among the represented biological situations. Furthermore, new graphical interfaces to access sequences belonging to specific metabolic pathways will add value to the already available links to KEGG [24].

## Conclusions

The version VI of ESTree db offers the possibility to access data concerning peach gene expression in an easy and complete way. ESTs multiple origins with respect to tissues and developmental stages allow a broad study of gene expression in a fruit tree. Ontologies analysis allows the comparison of the transcriptomes from different biological sets, and the creation of expression profiles, to be used in the study of patterns of gene expression (Vecchietti *et al*., in preparation).

Additional features of version VI of the ESTree db are the increased, more user-friendly, data retrieval systems according to custom-selected aspects implemented via the web interface. The continuous data production by the ESTree Interuniversitary Centre units will result in a growth of the ESTree db, that will be updated and upgraded according to the future peach genomic projects requirements.

## Availability and requirements

The ESTree db is available without restrictions at the following URL: .

## Competing interests

The authors declare that they have no competing interests.

## Authors' contributions

BL participated to library preparation, defined the pipeline structure and parameters and drafted the manuscript. AC structured the database and wrote all the accessory programs. AV was responsible for library preparation and sequence management. IM was the match point between the ESTree db and GRID technology. FB was responsible for the ESTree mapping project. LM coordinated the integration of bioinformatical resources. AS participated in the design of the study and critically revised the manuscript. CP guided and coordinated the execution of the project. All authors read and approved the final manuscript.
